# Quantification of the cerebral pressure–flow relationship directional sensitivity: Reliability of shorter repeated squat–stand durations

**DOI:** 10.1113/EP092357

**Published:** 2025-06-26

**Authors:** Lawrence Labrecque, Marc‐Antoine Roy, Shahrzad Soleimani Dehnavi, Mahmoudreza Taghizadeh, Joel S. Burma, Jonathan D. Smirl, Patrice Brassard

**Affiliations:** ^1^ Department of Kinesiology, Faculty of Medicine Université Laval Quebec City Quebec Canada; ^2^ Research center of the Institut universitaire de cardiologie et de pneumologie de Québec Quebec City Quebec Canada; ^3^ Sport Injury Prevention Research Centre, Faculty of Kinesiology University of Calgary Calgary Alberta Canada; ^4^ Concussion Research Laboratory, Faculty of Health and Exercise Science University of British Columbia Kelowna British Columbia Canada; ^5^ Human Performance Laboratory, Faculty of Kinesiology University of Calgary Calgary Alberta Canada; ^6^ Hotchkiss Brain Institute University of Calgary Calgary Alberta Canada; ^7^ Integrated Concussion Research Program University of Calgary Calgary Alberta Canada; ^8^ Alberta Children's Hospital Research Institute University of Calgary Calgary Alberta Canada; ^9^ Libin Cardiovascular Institute of Alberta University of Calgary Calgary Alberta Canada

**Keywords:** cerebral pressure–flow relationship, directional sensitivity, dynamic cerebral autoregulation

## Abstract

The magnitude of changes in middle cerebral artery mean blood velocity (MCAv) is attenuated when mean arterial pressure (MAP) increases compared with when MAP decreases. This directional sensitivity has been characterized using a time‐corrected ratio calculated on MCAv and MAP changes induced by repeated squat–stands (RSS) at 0.05 and 0.10 Hz for 300 s (∆MCAv_T_/∆MAP_T_). Herein, we examined the reliability of the metric within reduced RSS durations. Ninety‐nine (25 females) healthy human participants (26 ± 5 years) performed 300 s RSS at 0.05 Hz (20 s cycles) and 0.10 Hz (10 s cycles) while MCAv and MAP were measured continuously. The ∆MCAv_T_/∆MAP_T_ was calculated for each transition [increase (INC); decrease (DEC)] of MAP for 60, 120, 180, 240 and 300 s. A two‐way ANOVA was completed, and the absolute (Bland–Altman plot) and relative (coefficient of variation and intraclass correlation coefficient) reliability were calculated to compare shorter‐duration recordings with 300 s (reference standard). At 0.05 Hz, ∆MCAv_T_/∆MAP_T_ was similar between INC and DEC and comparable to the 300 s reference from 120 s onwards. At 0.10 Hz, ∆MCAv_T_/∆MAP_T_ was lower during INC (*p *< 0.0001). Bland–Altman plots indicated that differences trended towards zero (greater agreement) with increasing duration. Averaged coefficients of variation were <10% from 120 s (0.05 Hz) and 60 s (0.10 Hz) onwards. All intraclass correlation coefficients were >0.90 for recordings of ≥180 s in both frequencies. Although the 300 s reference is optimal, RSS duration could be shortened to 180 s, if needed, to identify this hysteresis‐like pattern reliably using ∆MCAv_T_/∆MAP_T_.

## Abstract (highlights)

- **What is the central question of this study?**
  We investigated whether a shorter duration of repeated squat–stands (RSS) can identify cerebral pressure–flow relationship directional sensitivity with similar reliability to the 300 s duration.
- **What is the main finding and its importance?**
  Findings indicate that 240 and 180 s recordings of RSS can be used to identify the cerebral pressure–flow relationship directional sensitivity reliably using our metric. Utilization of shorter periods of RSS could be beneficial when examining clinical populations or individuals with low cardiorespiratory fitness who might be unable to sustain the full 5 min of RSS owing to high physical demand.

## 1 INTRODUCTION

Although the brain is a relatively small portion of the total human body mass (2%–3%), it receives 15%–20% of total cardiac output and consumes ∼20% of the oxygen supply to the body (Kety & Schmidt, 1945). Maintaining the regulatory control of the blood supply to the brain is therefore critically important. To do so, a myriad of regulatory mechanisms work together to ensure that the perfusion of the brain is adapted to its demand. One of the mechanisms to limit the rapid transmission of arterial blood pressure variations to the cerebral blood supply is known as dynamic cerebral autoregulation (dCA) (Brassard et al., 2023; Claassen et al., 2021). The repeated squat–stand (RSS) manoeuvre is one of the most widely used methodologies to examine the cerebral pressure–flow relationship, owing to its ability to produce large and reproducible arterial blood pressure oscillations in healthy, able‐bodied individuals (Burma et al., 2024; Smirl et al., 2015). This technique provides the greatest reliability and reproducibility for quantifying the linear relationship between cerebral blood velocity (CBv) and mean arterial pressure (MAP) within the frequency domain using transfer function analysis (TFA) (Claassen et al., 2016; Panerai, Brassard et al., 2022; Smirl et al., 2015). Most often, 5 min RSS are completed at 0.05 Hz (10 s standing, 10 s squatting) and 0.10 Hz (5 s standing, 5 s squatting), because they occur within frequency bands where cerebral autoregulation is thought to be most active (Claassen et al., 2016; Panerai, Brassard et al., 2022). This 5 min minimum recording length has been proposed to stabilize TFA estimates for spontaneous (resting) oscillations in the low‐frequency band (i.e., as data length increases, the average fluctuation of a dCA measure becomes stochastically smaller until they reach the point of stability, defined as the minimal data length such that the average fluctuation of a dCA measure remains within a selected corridor of stability, representing the corridor around zero where the deviations of the average fluctuation of a dCA measure are tolerated) (Mahdi et al., 2017) and provide adequate frequency resolution (Claassen et al., 2016; Panerai, Brassard et al., 2022). This duration was therefore maintained as a standard when forcing oscillations using RSS (Claassen et al., 2016; Panerai, Brassard et al., 2022). Unfortunately, some testing situations might result in interrupted or inconsistent data collection, leading to good‐quality recordings (i.e., absence of non‐physiological artefacts) shorter than 5 min. For instance, technical issues with recording devices can arise during an experiment. In addition, the ability to use shorter RSS durations to quantify dCA would be highly beneficial when examining clinical populations or individuals with low cardiorespiratory fitness who might be unable to sustain the full 5 min of RSS owing to high physical demand.

Some studies examined the impact of shortening the duration of recordings when using TFA metrics during spontaneous (Olsen et al., 2024) and RSS‐driven oscillations (Barnes et al., 2018; Burma, Miutz et al., 2021). At 0.05 Hz, Barnes et al. (2018) established that coherence is already >0.90 after only three RSS or 60 s, which is shorter in duration than the minimal value recommended in the Cerebrovascular Research Network (CARNet) White Papers for reliable TFA estimates (i.e., phase and gain) (Claassen et al., 2016; Panerai, Brassard et al., 2022). When assessing the reliability of all TFA metrics at 0.05 and 0.10 Hz RSS, Burma, Miutz et al. (2021) also concluded that although 300 s (5 min) duration was ideal, RSS duration could be reduced to 240 s (4 min) and even 180 s (3 min) if some covariates [partial pressure of end‐tidal carbon dioxide ($P_{\mathrm{ET},CO_{2}}$), MAP, respiratory rate, etc.] were controlled for. Findings from these two studies support the possibility of shortening the RSS duration in certain experimental conditions, although both sets of authors concluded that a full RSS sequence of ≥5 min should be recorded whenever possible, with shorter recordings being used as a salvaging technique as required (Barnes et al., 2018; Burma, Miutz et al., 2021).

Accumulating evidence describes the presence of a directional sensitivity in the human cerebral pressure–flow relationship (Abbariki et al., 2022; Labrecque, Smirl, et al., 2021; Labrecque et al., 2022, 2024; Panerai et al., 2018; Panerai, Barnes, et al., 2022; Roy et al., 2022), referencing the attenuated CBv elevation when MAP increases acutely compared with the decline in CBv when MAP decreases acutely. This hysteresis‐like pattern could be a protective mechanism for the brain and its vasculature (Monro–Kellie doctrine), which is nested in a rigid bone cavity, requiring added protection against MAP surges (Wilson, 2016). A better understanding of this phenomenon is of the utmost importance, considering that numerous physiological (rapid eye movement sleep and exercise) and pathological conditions associated with MAP surges could have important cerebrovascular consequences. It is therefore crucial to adapt our analytical and modelling techniques to quantify dCA in health and disease.

Although it is the most widely used approach to quantify dCA, TFA has a major limitation, which is that it does not consider the direction of CBv and MAP changes (Kostoglou et al., 2024). In contrast, directional sensitivity assessments can be used to address this limitation. The directional sensitivity in the cerebral pressure–flow relationship has been identified using various analytical techniques (Brassard et al., 2017; Panerai et al., 2018; Panerai, Barnes, et al., 2022; Schmidt et al., 2009; Tzeng et al., 2010). Recently, we suggested the use of a new type of analysis in the time domain. We elaborated a time‐adjusted ratio (ΔMCA_T_/ΔMAP_T_) on CBv and MAP oscillations induced by RSS (Labrecque, Smirl, et al., 2021) and oscillatory lower‐body negative pressure (Labrecque et al., 2024). In addition to being simple to compute, our metric is processed on induced MAP oscillations without using a separate baseline. Importantly, this ratio metric can be calculated in absolute values (ΔMCAv_T_/ΔMAP_T_), but also in relative values (%MCAv_T_/%MAP_T_), permitting the comparison of different groups or anatomical regions with differing baseline characteristics (Labrecque et al., 2022). Using this metric, we reported directional sensitivity with 0.10 Hz, but not 0.05 Hz, oscillations induced by RSS (Labrecque et al., 2022; Labrecque, Smirl, et al., 2021) and oscillatory lower‐body negative pressure (Labrecque et al., 2024). Of note, although some studies have also reported the absence of a directional sensitivity at 0.05 Hz (Aaslid et al., 2007; Katsogridakis et al., 2017), others did (Panerai et al., 2018). Several reasons, discussed by Labrecque et al. (2024), could explain these discrepancies. Previously, it was demonstrated that this analytical approach is reliable over the 5 min RSS duration (Labrecque, Smirl, et al., 2021). However, it remains to be determined whether the metric can identify directional sensitivity when shorter RSS durations are used. Moreover, it is important to determine whether our metric would still be reliable in the eventuality of a shortening in RSS duration.

The first aim of this analysis was to examine the capability of our ΔMCAv_T_/ΔMAP_T_ and %MCAv_T_/%MAP_T_ metrics to reproduce our directional sensitivity findings (Abbariki et al., 2022; Labrecque et al., 2022, 2024; Labrecque, Smirl, et al., 2021; Roy et al., 2022), with shorter RSS duration. The second aim was to evaluate the reliability of our ΔMCAv_T_/ΔMAP_T_ and %MCAv_T_/%MAP_T_ metrics during various recording durations of RSS at both 0.05 and 0.10 Hz. To do so, 60, 120, 180 and 240 s recordings were compared with the reference 300 s RSS duration on the largest sample size to date. We hypothesized that ΔMCAv_T_/ΔMAP_T_ and %MCAv_T_/%MAP_T_ metrics would be lower during acute increases, in comparison with acute decreases, in MAP for all durations at 0.10 Hz, but not 0.05 Hz. Consistent with the broader dCA literature with respect to TFA, it was also hypothesized that ΔMCAv_T_/ΔMAP_T_ and %MCAv_T_/%MAP_T_ would be reliable for 180 and 240 s durations when compared with the 300 s reference standard in both MAP directions and RSS frequencies (Burma, Miutz, et al., 2021).

## 2 MATERIALS AND METHODS

### 2.1 Ethical approval

This secondary analysis includes healthy participants who had previously been involved in research studies at one of three institutions (Université Laval, Canada; University of British Columbia, Canada; University of Calgary, Canada). The original research studies were approved by the *Comité d’éthique de la recherche de l'Institut universitaire de cardiologie et de pneumologie de Québec* (CER: 2013–2092 and 2015–2465), the University of British Columbia Clinical Review Ethical Board (CER: H11‐02576, H11‐03287 and H16‐00507) and the Conjoint Health Research Ethics Board at the University of Calgary (REB20‐1662 and REB20‐2112) according to the principles established by the *Declaration of Helsinki* (except for registration in a database). Of the 99 participants included in the current analysis, 28 participants came from studies that examined the effect of cardiorespiratory fitness (Labrecque et al., 2017), biological sex (Labrecque et al., 2019), habitual endurance or resistance exercise (Perry et al., 2019; Roy et al., 2022) and high‐intensity interval training protocols (Drapeau et al., 2019) on the cerebral pressure–flow relationship. Eighteen participants were from the control conditions of a study examining the effect of moderate‐intensity continuous exercise training and high‐intensity interval training on multiple cerebrovascular (Burma, Copeland, Macaulay, Khatra, & Smirl, 2020a, 2021, 2020, 2020), cardiovascular (Burma, Copeland, Macaulay, Khatra, & Smirl, 2020b) and visual parameters (Burma, Copeland, et al., 2021). Twenty‐four participants came from a study aiming to identify the impact of added resistance to RSS on the cerebral pressure–flow relationship over the cardiac cycle and between biological sexes (Newel et al., 2022). Fourteen participants were from a study investigating the dynamic cerebral pressure–flow relationship in lowlanders and high‐altitude natives (Smirl, Lucas, et al., 2014). Five participants were from a study investigating the influence of cerebrovascular resistance on the cerebral pressure–flow relationship (Smirl, Tzeng, et al., 2014). Finally, 10 participants were control subjects from a study examining the regulation of cerebral blood flow in long‐term heart transplant recipients (Burma, Kennedy, et al., 2021; Smirl, Haykowsky, et al., 2014). None of the participants was included in more than one study.

### 2.2 Study design

Participants included were selected among these previous studies according to the following criteria: aged from 18 to 45 years old, healthy (no cardiovascular, pulmonary or cerebrovascular disease), and having high‐quality MAP and CBv recordings in both 0.05 and 0.10 Hz RSS. A total of 99 participants were included (74 males; age, 26 ± 5 years; body mass index, 25 ± 4 kg/m^2^). No participant was taking any medication, apart from two females who were taking oral contraceptives continuously for >1 year and one female who was fitted with an intra‐uterine device. All other females were tested during their early follicular phase or the mid‐late follicular phase (days 0–10) of their menstrual cycle.

### 2.3 Experimental protocol

Participants arrived at the laboratory at ∼08.00 h. All participants were requested to abstain from food for 2 h, exercise for ≥6 h, and drinking alcohol or caffeine for ≥8 h before the beginning of the recording period. An initial 5 min standing baseline rest was achieved by all participants. Participants then performed RSS for ≥300 s (5 min) at frequencies of 0.05 Hz (10 s squat, 10 s standing) and 0.10 Hz (5 s squat, 5 s standing) in a randomized order. There was ≥10 min of standing between each RSS session. A familiarization process for the RSS movement was completed initially by mimicking the experimenter before the recorded experimentation started. During recording, experimenters ensured that speed and depth (back knee angle of 90°) of transitions were similar during the whole RSS protocol and for each participant (Smirl et al., 2015).

### 2.4 Instrumentation

Arterial blood pressure was measured using finger arterial clamping complemented with a height correction unit (Finometer Pro, Finapres Medical Systems, Amsterdam, The Netherlands; or Nexfin, Edwards Lifesciences, Irvine, CA, USA). Heart rate was measured using a three‐lead ECG. Middle cerebral artery blood velocity (MCAv) was monitored using transcranial Doppler ultrasonography (Spencer Technologies, Seattle, WA, USA; or Doppler Box, Compumedics DWL, San Juan Capistrano, CA, USA) on the temporal window. The MCAv is used as a surrogate for quantifying cerebral blood flow (Skow et al., 2022). The MCA was identified using a standardized protocol (Willie et al., 2011). Once the MCA was identified, a probe was fixed with a headband or a specialized fixation helmet (DiaMon, Compumedics DWL) using adhesive ultrasonic gel. Breath‐by‐breath recording of $P_{\mathrm{ET},CO_{2}}$ was completed in 92 participants during standing baseline and both RSS sequences through a gas analyser equipped with fast‐responding oxygen and carbon dioxide sensors and a pneumotach (Breezesuite, MedGraphics Corp., MN, USA; Ultima CardiO2 Gas Exchange Analysis System, MGC Diagnostics, MN, USA; ML206, ADInstruments, Colorado Springs, CO, USA) calibrated to known gas concentrations following the manufacturer's instructions before each evaluation. The gas analyser was adjusted for daily room temperature, ambient atmospheric pressure and percentage of humidity.

All data were sampled simultaneously at 1000 Hz with an analog‐to‐digital converter (Powerlab 16/30 ML880, ADInstruments) and saved for later analysis using commercially available software (LabChart v.7.1, ADInstruments). To account for established delays in our blood pressure‐monitoring devices, we applied a −250 ms (Nexfin) and −1000 ms (Finometer) time shift to ensure they were time aligned with transcranial Doppler ultrasound, ECG and respiratory signals. Continuous MAP and mean MCAv traces were obtained by calculating the average raw arterial blood pressure and MCAv over each cardiac cycle. These calculated signals were not interpolated or resampled. Further analyses (see Section 2.5) were completed on those signals.

### 2.5 Data analysis

**FIGURE 1:**
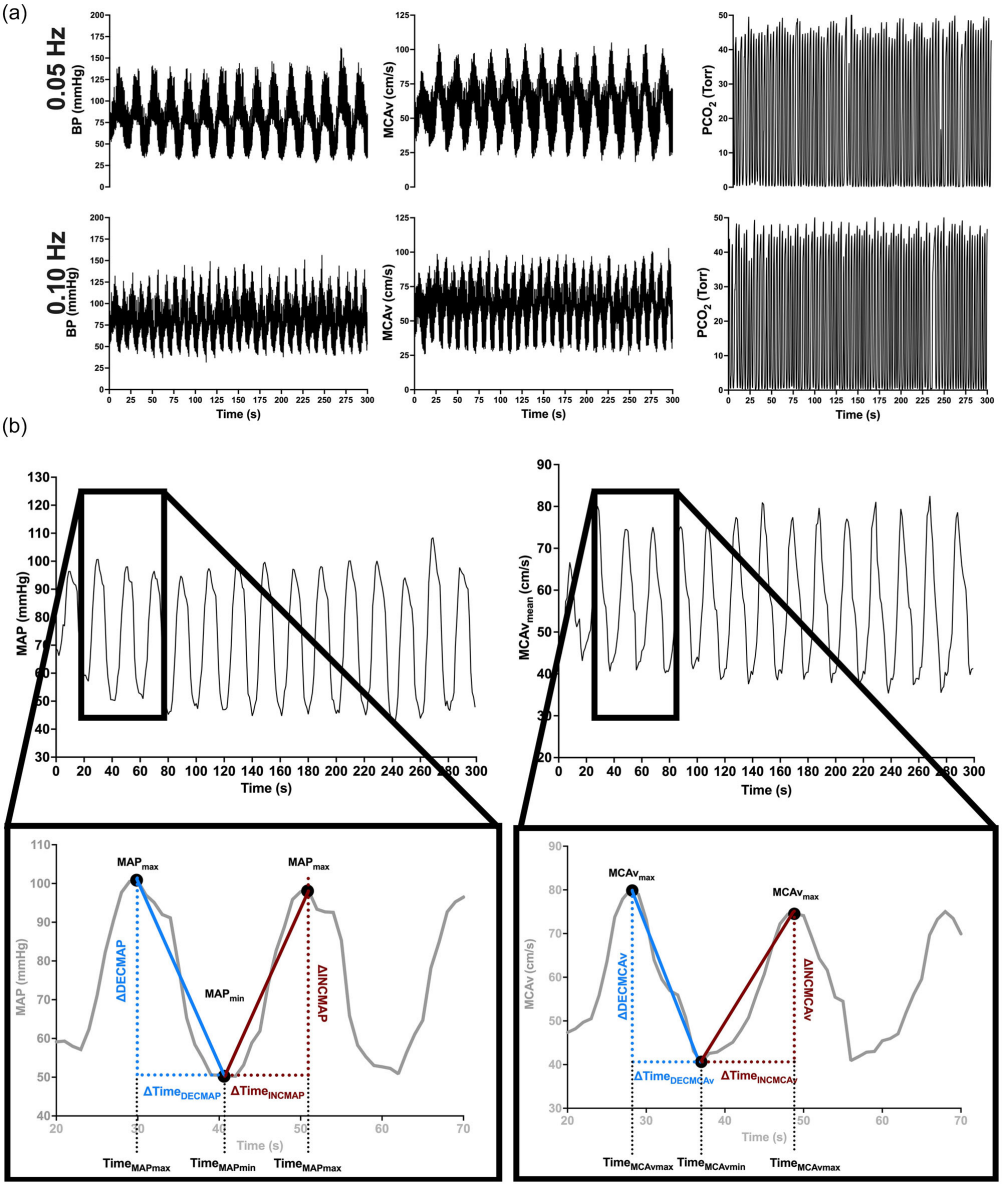
(a) Raw traces of BP, MCAv and $P_{CO_{2}}$ at 0.05 and 0.10 Hz RSS. (b) Schematic traces of MAP and MCAv during a RSS performed at 0.05 Hz. Enlarged regions depict how the variables were used to calculate ∆MCAv_T_/∆MAP_T_. Blue colour indicates variables used to calculate ∆MCAv_T_/∆MAP_T_^DEC^. Red colour indicates variables used to calculate ∆MCAv_T_/∆MAP_T_^INC^. Figure used and modified with permission from P. Brassard (Labrecque, Smirl, et al., 2021). Abbreviations: BP, blood pressure; MAP, mean arterial pressure; MAP_max_, maximum MAP value; MAP_min_, minimum MAP value; MCAv, middle cerebral artery blood velocity; MCAv_max_, maximum MCAv value; MCAv_min_, minimum MCAv value; $P_{CO_{2}}$, partial pressure of carbon dioxide; RSS, repeated squat–stands; Time_MAPmax_, time at maximum MAP; Time_MAPmin_, time at minimum MAP; Time_MCAvmax_, time at maximum MCAv; Time_MCAvmin_, time at minimum MCAv; ∆DECMAP, absolute change in MAP during acute decrease in MAP; ∆INCMAP, absolute change in MAP during acute increase in MAP; ∆DECMCAv, absolute change in MCAv during acute decrease in MAP; ∆INCMCAv, absolute change in MCAv during acute increase in MAP.

Baseline resting values in the standing position were averaged over the last 30–60 s before the onset of the first set of the RSS. To characterize the directional sensitivity in the cerebral pressure–flow relationship in response to transient increases and decreases in MAP, we calculated a time‐adjusted ratio between MCAv and MAP changes for each RSS transition in each MAP direction (Labrecque, Smirl, et al., 2021). Visual inspection was achieved initially to discard any unstable or atypical oscillations at the beginning of RSS. Absolute changes in MCAv (ΔMCAv) and MAP (ΔMAP) during each acute increase in MAP (INC) were calculated as the difference between maximal values and the preceding minimum for each transition. Absolute ΔMCAv and ΔMAP during each acute decrease in MAP (DEC) were calculated as the difference between the minimum and the preceding maximum. Time intervals (in seconds) for MCAv (Δtime MCAv) and MAP (Δtime MAP) changes were also calculated. To do so, the difference between the time at maximum value and the time at minimum value for each variable during INC for each transition was calculated. Likewise, the difference between the time at minimum value and the time at maximum value was calculated to obtain the time interval during DEC for each transition of both MAP and MCAv. The rate of change in MAP and MCAv was then calculated as ΔMAP/Δtime MAP and ΔMCAv/Δtime MCAv, respectively, for each transition in both MAP directions. For a visual representation of how these measures are calculated, we direct the reader to Figure 1. Finally, to characterize the cerebral pressure–flow relationship in response to INC and DEC, we calculated a time‐adjusted ratio between MCAv and MAP changes for each RSS transition in each MAP direction as previously described (Labrecque, Smirl, et al., 2021). Of note, all values that were >3SD from the mean were considered artefacts and removed from the analysis. All data sampling was initiated from the start of the data‐collection period (from 0 to 60 s, 0 to 120 s, 0 to 180 s, 0 to 240 s and 0 to 300 s). All the calculated values obtained for each RSS transition were then averaged over each RSS duration of interest (300 s or 5 min, 240 s or 4 min, 180 s or 3 min, 120 s or 2 min and 60 s or 1 min) in both MAP directions (INC and DEC) at both RSS frequencies (0.05 and 0.10 Hz).

The ∆MCAv_T_/∆MAP_T_ during DEC was calculated between a maximum and the following minimum as follows:

(1.1) $${\frac{\Delta \mathrm{MCA}v_{T}}{\Delta \mathrm{MA}P_{T}}}^{\mathrm{DEC}}=\frac{\frac{\Delta \mathrm{DECMCAv}}{\Delta \mathrm{Tim}e_{\mathrm{DECMCAv}}}}{\frac{\Delta \mathrm{DECMAP}}{\Delta \mathrm{Tim}e_{\mathrm{DECMAP}}}}=\frac{\frac{\left(\mathrm{MCA}v_{\min}-\mathrm{MCA}v_{\max}\right)}{\left(\mathrm{Tim}e_{\mathrm{MCAvmin}}-\mathrm{Tim}e_{\mathrm{MCAvmax}}\right)}}{\frac{\left(\mathrm{MA}P_{\min}-\mathrm{MA}P_{\max}\right)}{\left(\mathrm{Tim}e_{\mathrm{MAPmin}}-\mathrm{Tim}e_{\mathrm{MAPmax}}\right)}}$$

Where ∆DECMCAv is the absolute change in MCAv during DEC, ∆DECMAP is the absolute change in MAP during DEC, ΔTime_DECMCAv_ is the time interval of MCAv during DEC, ΔTime_DECMAP_ is the time interval of MAP during DEC, ΔMCAv_T_ is the rate of change in MCAv during DEC, and ΔMAP_T_ is the rate of change in MAP during DEC.

The ∆MCAv_T_/∆MAP_T_ during INC was calculated between a minimum and the following maximum as follows:

(1.2) $${\frac{\Delta \mathrm{MCA}v_{T}}{\Delta \mathrm{MA}P_{T}}}^{\mathrm{INC}}=\frac{\frac{\Delta \mathrm{INCMCAv}}{\Delta \mathrm{Tim}e_{\mathrm{INCMCAv}}}}{\frac{\Delta \mathrm{INCMAP}}{\Delta \mathrm{Tim}e_{\mathrm{INCMAP}}}}=\frac{\frac{\left(\mathrm{MCA}v_{\max}-\mathrm{MCA}v_{\min}\right)}{\left(\mathrm{Tim}e_{\mathrm{MCAvmax}}-\mathrm{Tim}e_{\mathrm{MCAvmin}}\right)}}{\frac{\left(\mathrm{MA}P_{\max}-\mathrm{MA}P_{\min}\right)}{\left(\mathrm{Tim}e_{\mathrm{MAPmax}}-\mathrm{Tim}e_{\mathrm{MAPmin}}\right)}}$$

Where ∆INCMCAv is the absolute change in MCAv during INC, ∆INCMAP is the absolute change in MAP during INC, ΔTime_INCMCAv_ is the time interval of MCAv during INC, ΔTime_INCMAP_ is the time interval of MAP during INC, ΔMCAv_T_ is the rate of change in MCAv during INC, and ΔMAP_T_ is the rate of change in MAP during INC.

Given that baseline values can be influenced by biological sex, age and cardiorespiratory fitness, we included a calculation using relative values (%MCAv_T_/%MAP_T_), where the minimum value for each respective squat–stand cycle was considered the ‘baseline’ for each INC and DEC. Therefore, the INC and DEC are comparable even if the MAP direction, and therefore the starting point, is different (Labrecque, Smirl, et al., 2021).

The %MCAv_T_/%MAP_T_ during DEC was calculated between a maximum and the following minimum as follows:

(2.1) $${\frac{\%\mathrm{MCA}v_{T}}{\%\mathrm{MA}P_{T}}}^{\mathrm{DEC}}=\frac{\frac{\%\mathrm{DECMCAv}}{\Delta \mathrm{Tim}e_{\mathrm{DECMCAv}}}}{\frac{\%\mathrm{DECMAP}}{\Delta \mathrm{Tim}e_{\mathrm{DECMAP}}}}=\frac{\frac{\left(\frac{\mathrm{MCA}v_{\min}-\mathrm{MCA}v_{\max}}{\mathrm{MCA}v_{\min}}\right)}{\left(\mathrm{Tim}e_{\mathrm{MCAvmin}}-\mathrm{Tim}e_{\mathrm{MCAvmax}}\right)}}{\frac{\left(\frac{\mathrm{MA}P_{\min}-\mathrm{MA}P_{\max}}{\mathrm{MA}P_{\min}}\right)}{\left(\mathrm{Tim}e_{\mathrm{MAPmin}}-\mathrm{Tim}e_{\mathrm{MAPmax}}\right)}}$$

Where %DECMCAv is the relative change in MCAv during DEC, %DECMAP is the relative change in MAP during DEC, %MCAv_T_ is the relative rate of change in MCAv, and %MAP_T_ is the relative rate of change in MAP during DEC.

The %MCAv_T_/%MAP_T_ during INC was calculated between a maximum and the previous minimum as follows:

(2.2) $${\frac{\%\mathrm{MCA}v_{T}}{\%\mathrm{MA}P_{T}}}^{\mathrm{INC}}=\frac{\frac{\%\mathrm{INCMCAv}}{\Delta \mathrm{Tim}e_{\mathrm{INCMCAv}}}}{\frac{\%\mathrm{INCMAP}}{\Delta \mathrm{Tim}e_{\mathrm{INCMAP}}}}=\frac{\frac{\left(\frac{\mathrm{MCA}v_{\max}-\mathrm{MCA}v_{\min}}{\mathrm{MCA}v_{\min}}\right)}{\left(\mathrm{Tim}e_{\mathrm{MCAvmax}}-\mathrm{Tim}e_{\mathrm{MCAvmin}}\right)}}{\frac{\left(\frac{\mathrm{MA}P_{\max}-\mathrm{MA}P_{\min}}{\mathrm{MA}P_{\min}}\right)}{\left(\mathrm{Tim}e_{\mathrm{MAPmax}}-\mathrm{Tim}e_{\mathrm{MAPmin}}\right)}}$$

Where %INCMCAv is the relative change in MCAv during INC, and %INCMAP is the relative change in MAP during INC, %MCAv_T_ is the relative rate of change in MCAv, and %MAP_T_ is the relative rate of change in MAP during INC.

The $P_{\mathrm{ET},CO_{2}}$ and breathing frequency data were averaged over each RSS duration at both frequencies.

### 2.6 Sample size calculation

This sample size was chosen according to multiple considerations. Initially, we ran an a priori power calculation from previous results published by our group (α = 0.05, β = 0.90) (Labrecque, Smirl, et al., 2021), where we observed that ∆MCAv_T_/∆MAP_T_^INC^ is lower than ∆MCAv_T_/∆MAP_T_^DEC^ at 0.10 Hz RSS (0.91 ± 0.3 vs. 1.01 ± 0.4 cm/s/mmHg), which indicated that 13 participants would be sufficient. However, considering the numerous analyses completed in this investigation, we adapted the sample size according to previous results by Burma, Miutz, et al. (2021), who ran a similar analysis using TFA metrics with 70 participants. To do so, we used a sample size calculator specifically for reliability studies using intraclass correlation coefficients (ICCs) (Arifin, 2018). Using a minimum acceptable reliability of 0.80 (which is greater than the 0.75 lower margin for a good reliability), an expected reliability of 0.90 (based on the findings by Burma, Miutz, et al., 2021), a significance level (α) of 0.05, a power (β) of 95%, and the minimal three repetitions per participant (*k*), we estimated that a minimal sample size of 68 participants would be necessary (Arifin, 2018). Of note, the lower number of repetitions used in our ICC calculations was considered because increasing the number of repetitions usually leads to lower sample sizes being required. Therefore, our sample size would have been underestimated. Owing to the greater variability of our ratio metric, we considered that increasing the sample size would benefit our analysis and provide the opportunity to characterize the directional sensitivity in the cerebral pressure–flow relationship, using these proposed metrics, on the largest sample size to date.

### 2.7 Statistical analyses

Statistical analyses were performed with Prism (GraphPad v.10.2.0 335) and R v.4.4.0 (2024‐04‐24). The normality of distributions was tested using Shapiro–Wilk tests. To evaluate the presence of directional sensitivity in the cerebral pressure–flow relationship through different RSS durations, a two‐way repeated‐measures ANOVA (factors: MAP direction and RSS duration as repeated measures) was used at each RSS frequency. For each participant, the average over each duration was used in the analysis. Dunnett's multiple comparison test was completed to evaluate simple effects between the 300 s RSS duration and the other RSS durations in each MAP direction. The Friedman multiple comparison test was applied to compare the $P_{\mathrm{ET},CO_{2}}$ and breathing frequency of each duration with the 300 s RSS duration reference. To determine the reliability of our ratio metric within different RSS durations, repeatability was evaluated by quantifying the absolute (or agreement; Hartmann et al., 2023) and relative reliability between each RSS duration and the 300 s reference (Bartlett & Frost, 2008). Absolute reliability (or agreement) quantifies how close two measures are completed on the same participant (Bartlett & Frost, 2008). It therefore does not depend on the population heterogeneity. Relative reliability relates the amplitude of the measurement error to the intrinsic variability. In other words, a high relative reliability indicates that measurement errors are small relative to the real differences between participants (Bartlett & Frost, 2008; Hartmann et al., 2023). Relative reliability then refers to the ability of a score to detect differences between participants (Kottner & Streiner, 2011). In this study, a reliability assessment was chosen over a validity assessment because no gold standard yet exists to identify cerebral pressure–flow relationship directional sensitivity (Hartmann et al., 2023).

Absolute reliability was evaluated using Bland–Altman plots, where ΔMCAv_T_/ΔMAP_T_ and %MCAv_T_/%MAP_T_ for each shorter RSS duration and the 300  s reference were reported. The 95% limits of agreement (LoA) were also calculated and reported as a percentage relative to the mean differences between methods (Bartlett & Frost, 2008; Hartmann et al., 2023). Relative reliability analysis was performed using the coefficient of variation (CoV) and ICC. The CoVs were calculated between each shorter RSS duration and the 300 s reference. The ICCs were calculated within each RSS duration (to evaluate the reliability over 60, 120, 180, 240 and 300 s) and between each RSS duration and the 300 s reference standard (to evaluate the reliability of each shorter RSS duration versus the 300 s reference standard). Within‐duration ICCs and their 95% confidence intervals were calculated using the ‘psych’ package (v.2.4.3) based on a mean rating (0.05 Hz: *k* = 3, 6, 9, 12 and 15 for 60, 120, 180, 240 and 300 s, respectively; 0.10 Hz: *k* = 6, 12, 18, 24 and 30 for 60, 120, 180, 240 and 300 s, respectively), absolute‐agreement, two‐way mixed‐effects model. Specifically, within each RSS duration, we assessed the reliability of our ratio for each squat–stand repetition compared with the average of that duration (average rating). For instance, at 0.05 Hz, the 1 min ICCs compared the first three squat–stands, the 2 min ICCs used the first six, up to 5 min ICCs comparing all 15 squat–stand repetitions. At 0.10 Hz, the 1 min ICCs compared the first six squat–stands, twice that of 0.05 Hz. The number ‘*k*’ represents the number of squat–stands for a given duration, with the same time points from shorter durations (e.g., squats 1–3 for 1 min) being included at the start of longer durations (e.g., squats 1–15 for 5 min). This gives an idea of how reliable each time point (squat–stand) is compared to the average obtained from that RSS duration. Between‐duration ICCs and their 95% confidence intervals were calculated using the ‘psych’ package (v.2.4.3) based on a single‐rating (*k* = 5), absolute‐agreement, two‐way mixed‐effects model. ICC estimates and their respective 95% confidence intervals are usually defined as <0.50 (poor), 0.50–0.75 (moderate), 0.75–0.90 (good) and >0.90 (excellent) (Koo & Li, 2016), which have been used throughout this manuscript (Koo & Li, 2016). Of note, in the event of negative values (i.e., values calculated during acute decreases in MAP and MCAv), absolute values were used to carry out descriptive calculations and statistical analysis. Values of *p* < 0.05 were considered significant.

## 3 RESULTS

### 3.1 Participants’ demographic and resting values

Our sample, including 99 participants, was composed of 25 females and 74 males. These participants were recreationally active (*n* = 77) or endurance trained in various aerobic sports (*n* = 22). Resting $P_{\mathrm{ET},CO_{2}}$ was not recorded in five participants, and technical problems occurred in two participants. Therefore, the final sample size for resting $P_{\mathrm{ET},CO_{2}}$ was 92. Baseline characteristics and resting standing values are reported in Table 1. Baseline $P_{\mathrm{ET},CO_{2}}$ was slightly reduced from the averaged $P_{\mathrm{ET},CO_{2}}$ during RSS at 0.10 Hz (39.9 ± 4.0 vs. 40.6 ± 4.3 Torr; *p* = 0.011) but not at 0.05 Hz (39.9 ± 4.0 vs. 39.8 ± 4.1 Torr; *p* = 0.948).

**TABLE 1:** Baseline characteristics and resting seated values.

Characteristic	Value
Baseline characteristics	
*n*	99
Age, years	24 (19–41)
Height, m	1.75 ± 0.09
Body mass, kg	75 ± 14
Body mass index, kg/m^2^	24.6 ± 3.5
Resting values	
Heart rate, beats/min	73 ± 14
Mean arterial pressure, mmHg	90 ± 12
Middle cerebral artery mean blood velocity, cm/s	62 ± 12
Partial pressure of end‐tidal carbon dioxide, Torr^a^	40 ± 4

*Note*: Age is reported as the median (range). All other variables are reported as the mean ± SD.

^a^ *n* = 92.

### 3.2 $P_{\mathrm{ET},CO_{2}}$ and breathing frequency during RSS

Averaged $P_{\mathrm{ET},CO_{2}}$ during the 5 min squat–stand repetitions were slightly elevated for 0.10 Hz (0.05 Hz: 39.8 ± 4.1 Torr; 0.10 Hz: 40.6 ± 4.3 Torr; *p *< 0.001). The average within‐subject $P_{\mathrm{ET},CO_{2}}$ difference was 2.2 Torr (median, 1.6 Torr) between baseline and 0.05 Hz RSS, 2.6 Torr (median, 2.0 Torr) between baseline and 0.10 Hz, and 1.6 Torr (median, 1.1 Torr) between both frequencies. At 0.05 Hz, there was a RSS duration effect for $P_{\mathrm{ET},CO_{2}}$ (*p* < 0.0001; Table 2) and breathing frequency (*p* = 0.0083; Table 2). At 0.10 Hz, there was also a RSS duration effect for both $P_{\mathrm{ET},CO_{2}}$ (*p* < 0.0001; Table 2) and breathing frequency (*p* = 0.0007; Table 2). Multiple comparisons where differences were revealed between shorter RSS durations and the 300 s reference duration are indicated with an asterisk in Table 2. Of note, no participant had a breathing frequency of <0.10 Hz (6 breaths/min).

### 3.3 Absolute changes in MAP, MCAv, and their respective time intervals and rate of change across RSS durations

For absolute changes in MAP, there was an RSS duration effect at both frequencies [0.05 Hz: *F*(4,392) = 30.09; *p *< 0.0001; 0.10 Hz: *F*(4,392) = 99.36; *p *< 0.0001], a MAP direction effect at 0.05 Hz only [*F*(1,98) = 5.543; *p* = 0.021], and an interaction effect at both frequencies [0.05 Hz: *F*(4,392) = 3.059; *p* = 0.017; 0.10 Hz: *F*(4,392) = 11.34; *p *< 0.0001]. For absolute changes in MCAv, there were RSS duration [0.05 Hz: *F*(4,392) = 34.65; *p *< 0.0001; 0.10 Hz: *F*(4,392) = 99.81; *p *< 0.0001] and MAP direction [0.05 Hz: *F*(1,98) = 12.45; *p* = 0.001; 0.10 Hz: *F*(1,98) = 6.718; *p* = 0.011] effects at both frequencies, in addition to an interaction effect at 0.05 Hz only [*F*(4,392) = 8.067; *p *< 0.0001]. For time intervals of MAP, there was a MAP direction effect at 0.05 Hz [*F*(1,98) = 55.60; *p *< 0.0001] and 0.10 Hz [*F*(1,98) = 40.21; *p *< 0.0001], and an interaction effect at 0.10 Hz [*F*(1.924,188.6) = 8.839; *p* = 0.001]. For time interval of MCAv, there was a RSS duration effect at 0.10 Hz [*F*(4,392) = 4.859; *p* = 0.001] and an MAP direction [*F*(1,98) = 32.35; *p *< 0.0001], in addition to an interaction [*F*(4,392) = 3.652; *p* = 0.006] effect at 0.05 Hz. For the rate of change in MAP, there was an RSS duration [0.05 Hz: *F*(4,392) = 5.110; *p* < 0.001; 0.10 Hz: *F*(4,392) = 58.14; *p* < 0.0001] and an MAP direction [0.05 Hz: *F*(1,98) = 48.29; *p *< 0.0001; 0.10 Hz: *F*(1,98) = 31.42; *p *< 0.0001] effect at both frequencies. For the rate of change in MCAv, there was an RSS duration effect [0.05 Hz: *F*(4,392) = 38.16; *p *< 0.0001; 0.10 Hz: *F*(4,392) = 98.91; *p *< 0.0001] at both frequencies, and a MAP direction effect at 0.05 Hz [*F*(1,98) = 32.21; *p *< 0.0001]. Multiple comparisons where differences were revealed between shorter RSS durations and the 300 s reference duration are indicated with an asterisk in Table 3.

**TABLE 2:** End‐tidal partial pressure of carbon dioxide and breathing frequency for each repeated squat–stand duration at 0.05 Hz and 0.10 Hz.

Parameter	60 s	120 s	180 s	240 s	300 s	*p*‐Value
**0.05** **Hz**						
$P_{\mathrm{ET},CO_{2}}$, Torr	38.6 ± 6.0^a^	38.9 ± 6.1^a^	38.8 ± 6.4^a^	39.0 ± 6.2	39.1 ± 6.2	<0.0001
Breathing frequency, breaths/min	15.8 ± 3.8^a^	16.0 ± 3.9^a^	16.0 ± 4.1	16.1 ± 4.0	16.2 ± 4.0	0.0083
**0.10** **Hz**						
$P_{\mathrm{ET},CO_{2}}$, Torr	39.1 ± 6.1^a^	39.5 ± 6.1^a^	39.7 ± 6.1^a^	39.9 ± 6.2	40.0 ± 6.2	<0.0001
Breathing frequency, breaths/min	16.2 ± 4.2^a^	16.4 ± 4.2^a^	16.4 ± 4.1	16.5 ± 4.1	16.6 ± 4.1	0.0007

^a^ Values different from 300 s duration. Abbreviation: $P_{\mathrm{ET},CO_{2}}$, end‐tidal partial pressure of carbon dioxide.

### 3.4 Directional sensitivity of the cerebral pressure–flow relationship in the MCA across different durations of RSS

**FIGURE 2:**
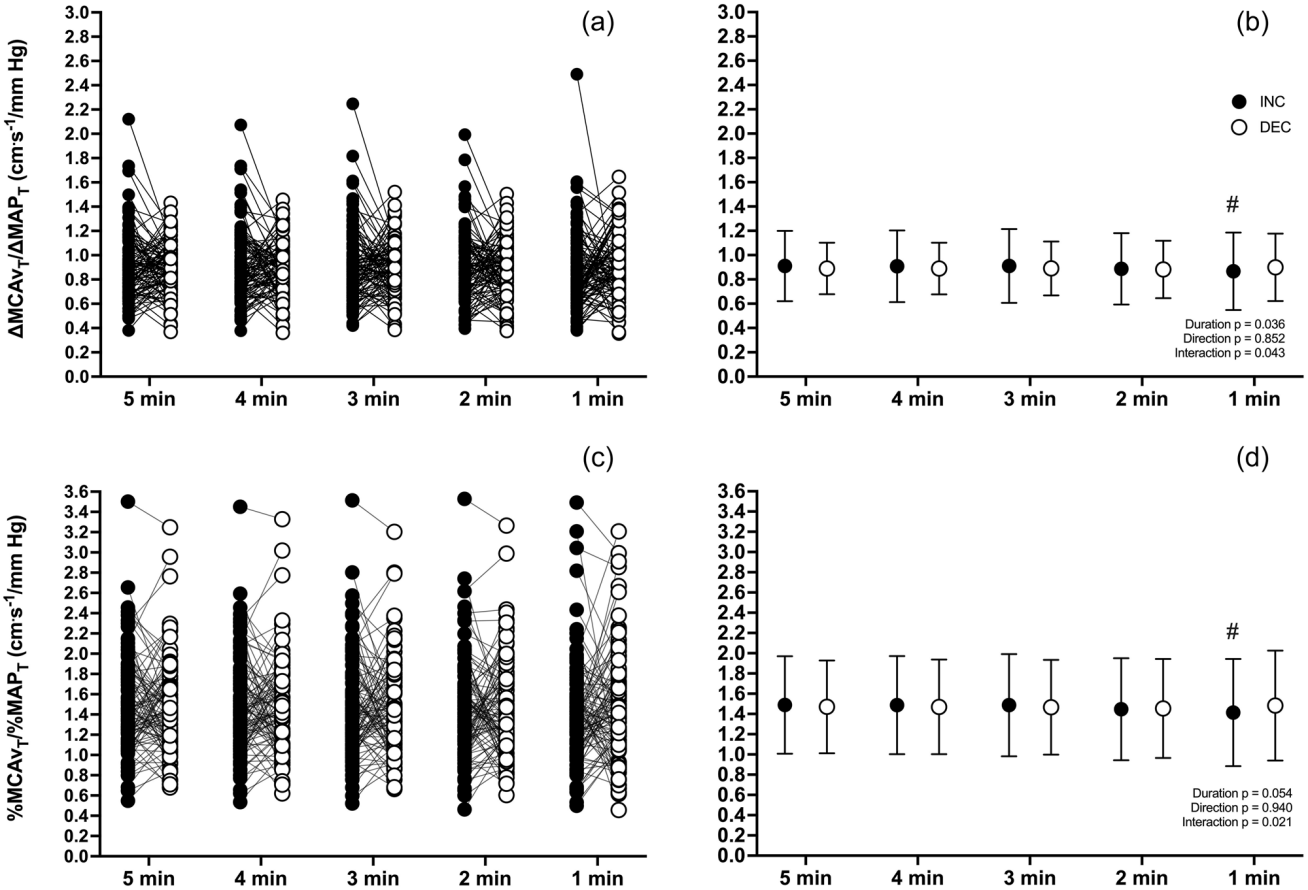
∆MCAv_T_/∆MAP_T_ and %MCAv_T_/%MAP_T_ during transient increases (INC; filled circles) and decreases (DEC; open circles) for all repeated squat–stand durations at 0.05 Hz. Left panels (a, c) represent individual values, and right panels (b, d) represent averaged values over the 5 min repeated squat–stand. Differences were assessed via a two‐way repeated‐measures ANOVA (factors: MAP direction and duration as repeated measures). ^#^Significant difference from corresponding value at 300 s. *n* = 99. Abbreviations: DEC, decrease; INC, increase; MAP, mean arterial pressure; MCA, middle cerebral artery.

For ∆MCAv_T_/∆MAP_T_ at 0.05 Hz, there was an RSS duration effect [*F*(4,392) = 3.598; *p* = 0.036] and an interaction effect [*F*(4,392) = 2.482; *p* = 0.0434], but no effect of MAP direction. Multiple comparisons revealed that only ∆MCAv_T_/∆MAP_T_^INC^ averaged over 60 s of RSS was different from the average over 300 s (*p* < 0.01). For %MCAv_T_/%MAP_T_, there was only an interaction effect [*F*(4,392) = 2.935; *p* = 0.021], and multiple comparisons also indicated that %MCAv_T_/%MAP_T_^INC^ averaged over 60 s of RSS was different from the average over 300 s (*p* = 0.003) (Figure 2).

**FIGURE 3:**
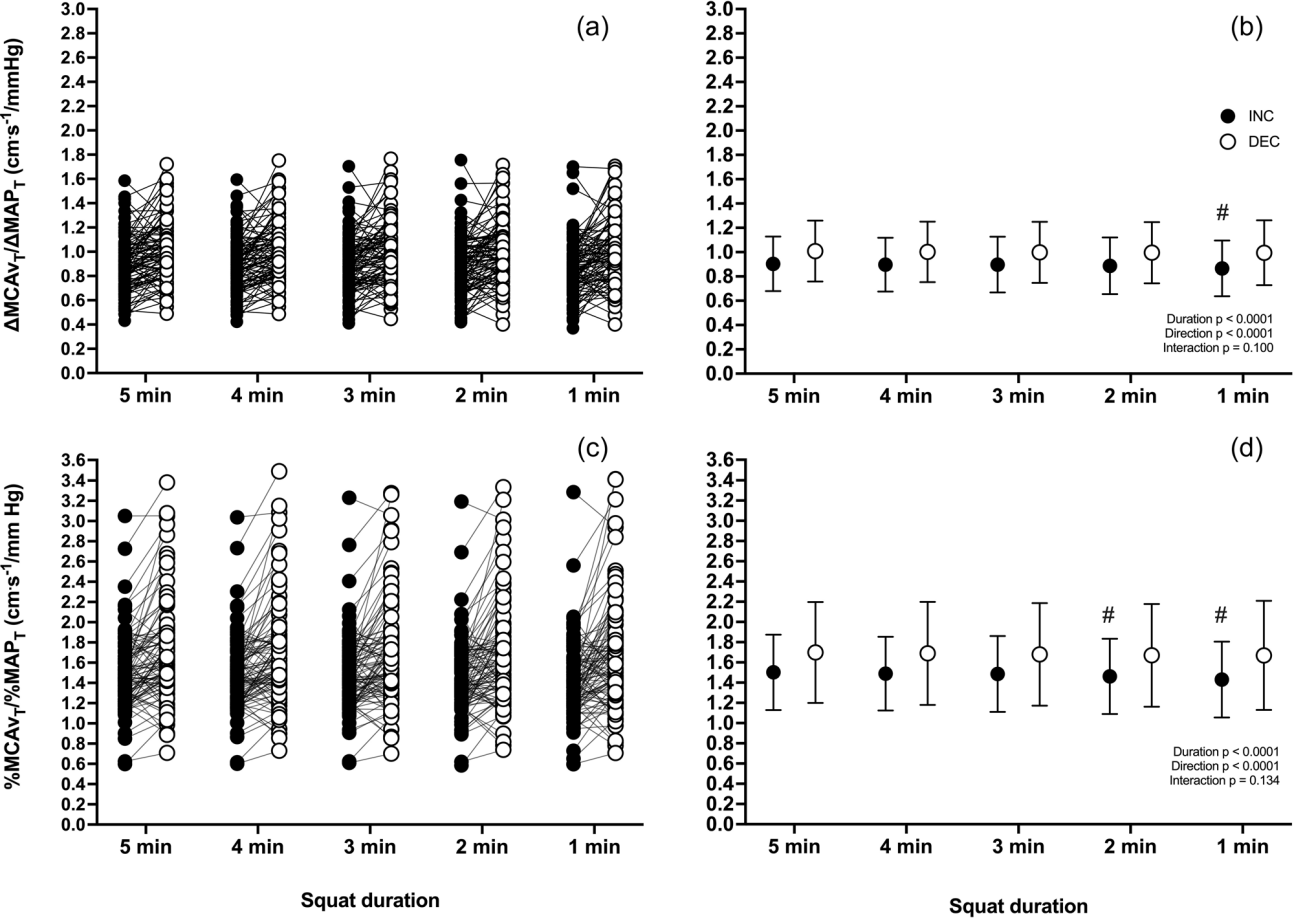
∆MCAv_T_/∆MAP_T_ and %MCAv_T_/%MAP_T_ during transient increases (INC; filled circles) and decreases (DEC; open circles) for all repeated squat–stand durations at 0.10 Hz. Left panels (a, c) represent individual values, and right panels (b, d) represent averaged values over the 5 min repeated squat–stand. Differences were assessed via a two‐way repeated‐measures ANOVA (factors: MAP direction and duration as repeated measures). ^#^Significant difference from corresponding value at 300 s. *n* = 99. Abbreviations: DEC, decrease; INC, increase; MAP, mean arterial pressure; MCA, middle cerebral artery.

For ∆MCAv_T_/∆MAP_T_ at 0.10 Hz, there was an RSS duration [*F*(4,302) = 7.513; *p *< 0.0001] and an MAP direction effect [*F*(1,98) = 25.37; *p *< 0.0001]. Multiple comparisons revealed that only ∆MCAv_T_/∆MAP_T_^INC^ averaged over 60 s of RSS was different from the average over 300 s (*p* ≤ 0.0001). For %MCAv_T_/%MAP_T_, there was also a RSS duration [*F*(4,302) = 8.965; *p *< 0.0001] and an MAP direction [*F*(1,98) = 29.04; *p *< 0.0001] effect. Multiple comparisons revealed that %MCAv_T_/%MAP_T_^INC^ averaged over 60 s (*p *< 0.0001) and 120 s (*p* = 0.0137) of RSS were different from the average over 300 s (Figure 3).

### 3.5 Absolute reliability of the ratio metric to identify directional sensitivity at different durations of RSS

**FIGURE 4:**
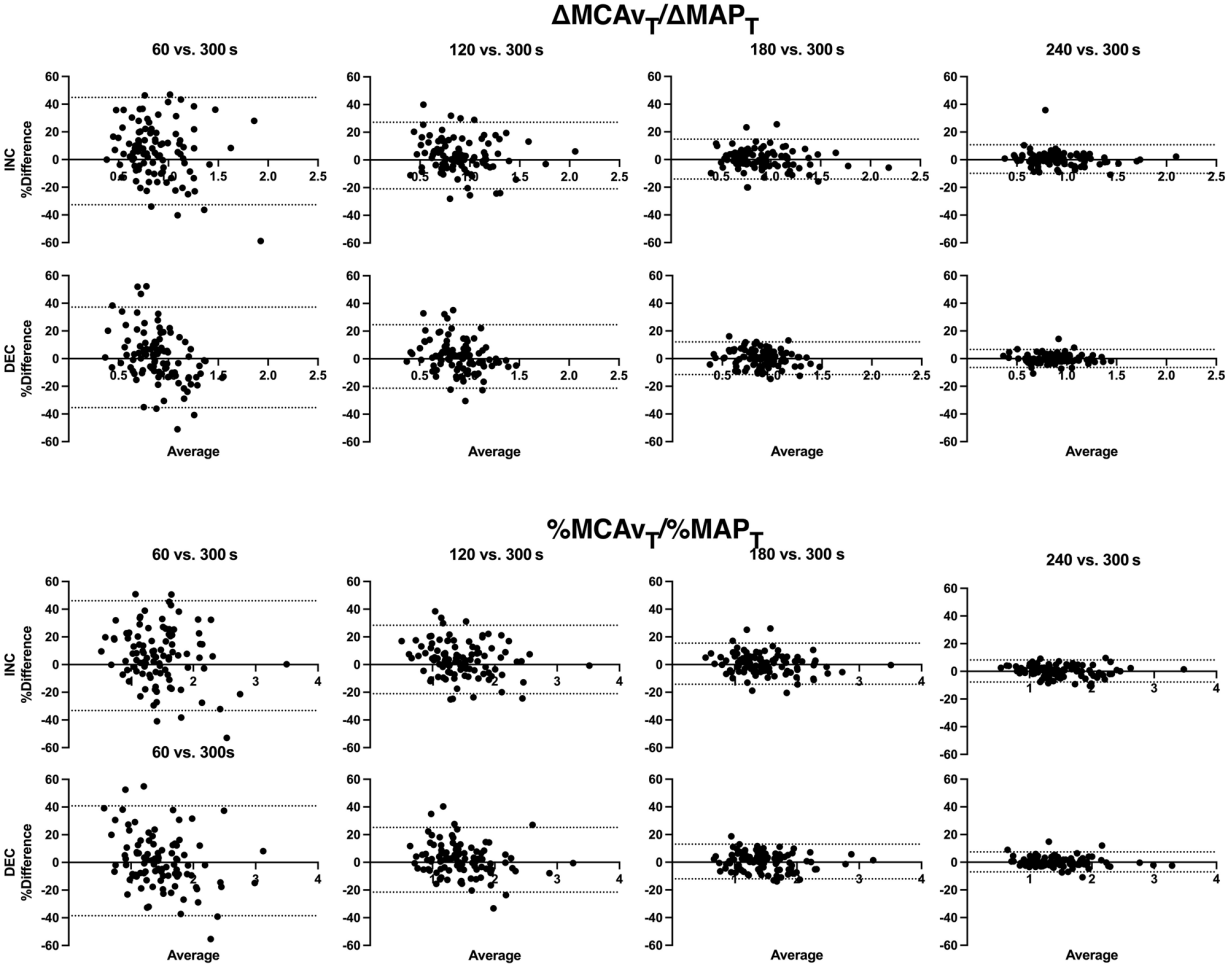
Bland–Altman plot with 95% LoA of ∆MCAv_T_/∆MAP_T_ and %MCAv_T_/%MAP_T_ at 0.05 Hz reported as the relative difference (as a percentage) between durations versus the average. Shorter durations are compared with the 300 s reference standard. *n* = 99. Abbreviation: LoA, limits of agreement.

**FIGURE 5:**
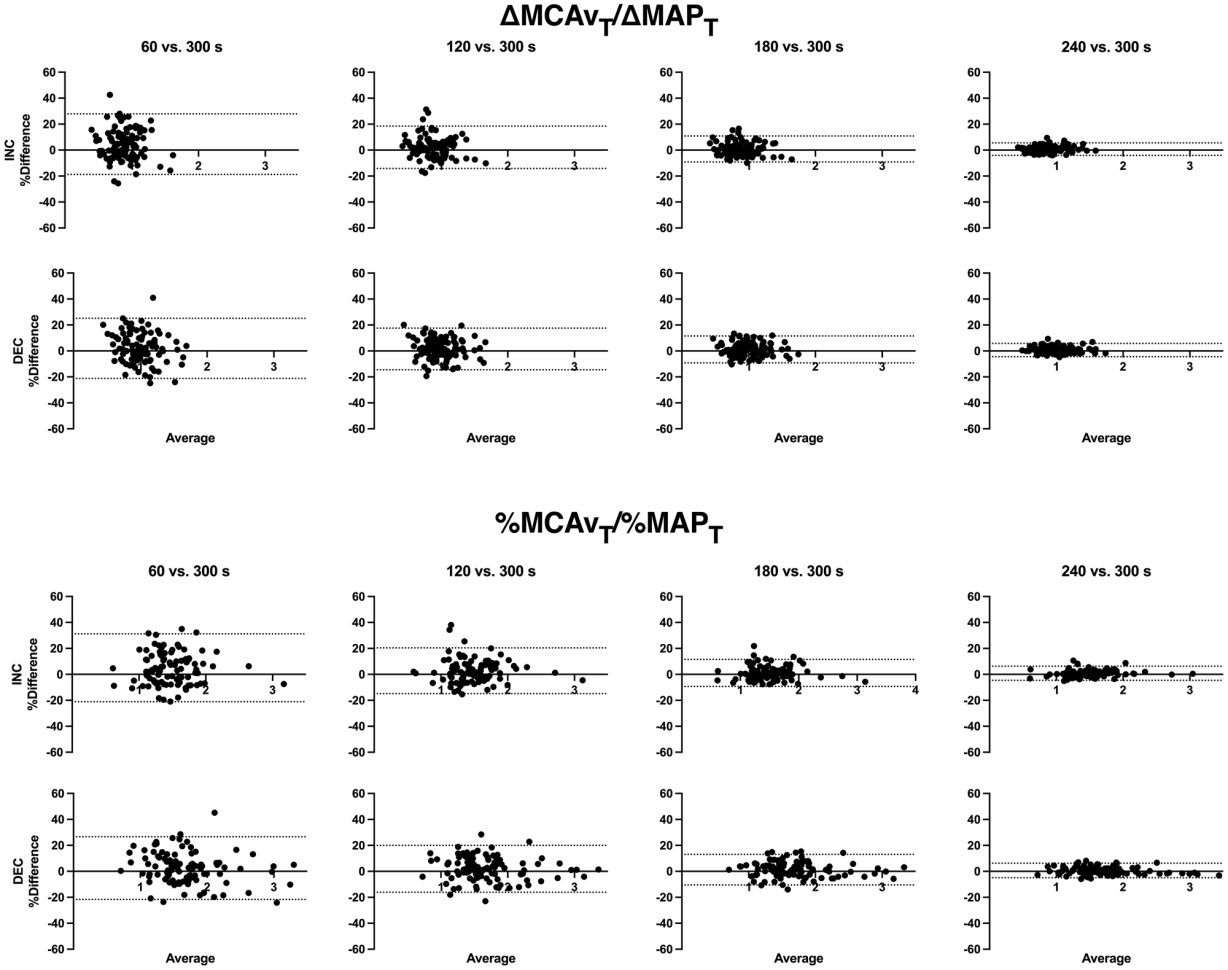
Bland–Altman plot with 95% LoA of ∆MCAv_T_/∆MAP_T_ and %MCAv_T_/%MAP_T_ at 0.10 Hz reported as the relative difference (as a percentage) between durations versus the average. Shorter durations are compared with the 300 s reference standard. *n* = 99. Abbreviation: LoA, limits of agreement.

Figures 4 and 5 depict Bland–Altman plots with 95% LoA for ∆MCAv_T_/∆MAP_T_ and %MCAv_T_/%MAP_T_ comparing 60, 120, 180 and 240 s with the reference 300 s standard RSS duration. As duration increased, plotted differences with the 300 s standard tended toward zero (more comparable) at both RSS frequencies.

### 3.6 Relative reliability of the ratio metric to identify directional sensitivity at different durations of RSS

For both ∆MCAv_T_/∆MAP_T_ and %MCAv_T_/%MAP_T_, averaged CoVs indicate an increasing relative reliability via a reduced CoV with increasing RSS duration. For MCAv_T_/∆MAP_T_^INC^ and MCAv_T_/∆MAP_T_^DEC^ at both frequencies, apart from MCAv_T_/∆MAP_T_^INC^ for 60 versus 300 s at 0.05 Hz, all averaged CoVs were <10%, which is indicative of a good reliability (Smirl et al., 2015). The finding was comparable for %MCAv_T_/%MAP_T_^INC^ and %MCAv_T_/%MAP_T_^DEC^ at both frequencies, where only two averaged CoVs values were slightly >10% (%MCAv_T_/%MAP_T_^INC^ and %MCAv_T_/%MAP_T_^DEC^ for 60 versus 300 s at 0.05 Hz). All these results are reported in Tables 4 and 5.

**FIGURE 6:**
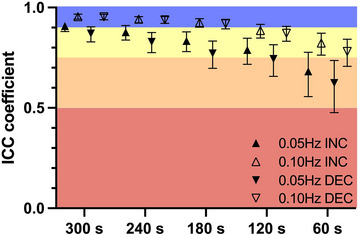
Within‐duration ICC of ∆MCAv_T_/∆MAP_T_ for 60, 120, 180, 240 and 300 s duration RSS. Excellent (>0.90), good (0.75–0.90), moderate (0.50–0.75) and poor (<0.50) ICC are differentiated by blue, yellow, orange and red zones, respectively. *n* = 99. Abbreviations: DEC, decrease; ICC, intraclass correlation coefficient; INC, increase; RSS, repeated squat–stands.

**FIGURE 7:**
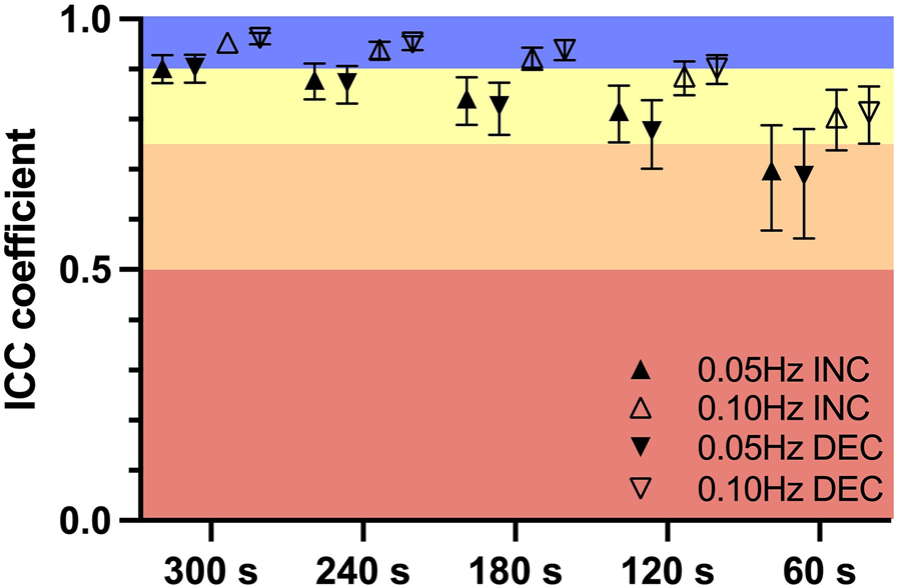
Within‐duration ICC of %MCAv_T_/%MAP_T_ for 60, 120, 180, 240 and 300 s duration RSS. Excellent (>0.90), good (0.75–0.90), moderate (0.50–0.75) and poor (<0.50) ICC are differentiated by blue, yellow, orange and red zones, respectively. *n* = 99. RSS, Abbreviations: DEC, decrease; ICC, intraclass correlation coefficient; INC, increase; repeated squat–stands.

Within‐duration ICCs are reported in Figures 6 and 7. At 0.05 Hz, within‐duration ICCs and their 95% CIs were 0.682 [0.556–0.777] (60 s), 0.789 [0.717–0.847] (120 s), 0.834 [0.780–0.879] (180 s), 0.877 [0.838–0.910] (240 s) and 0.908 [0.880–0.933] (300 s) for ∆MCAv_T_/∆MAP_T_^INC^ and 0.624 [0.476–0.736] (60 s), 0.744 [0.657–0.815] (120 s), 0.771 [0.697–0.833] (180 s), 0.829 [0.775–0.875] (240 s) and 0.870 [0.829–0.904] (300 s) for ∆MCAv_T_/∆MAP_T_^DEC^. Still at 0.05 Hz, within‐duration ICCs and their 95% CIs were 0.698 [0.578–0.788] (60 s), 0.816 [0.754–0.867] (120 s), 0.841 [0.789–0.884] (180 s), 0.879 [0.840–0.911] (240 s) and 0.902 [0.872–0.928] (300 s) for %MCAv_T_/%MAP_T_^INC^, and 0.687 [0.562–0.780] (60 s), 0.776 [0.701–0.838] (120 s), 0.826 [0.769–0.873] (180 s), 0.872 [0.831–0.906] (240 s) and 0.903 [0.873–0.929] (300 s) for %MCAv_T_/%MAP_T_^DEC^. At 0.10 Hz, within‐duration ICCs and their 95% CIs were 0.823 [0.763–0.872] (60 s), 0.885 [0.848–0.916] (120 s), 0.925 [0.902–0.945] (180 s), 0.942 [0.924–0.957] (240 s) and 0.955 [0.942–0.967] (300 s) for ∆MCAv_T_/∆MAP_T_^INC^, and 0.781 [0.707–0.842] (60 s), 0.872 [0.8032–0.906] (120 s), 0.919 [0.894–0.941] (180 s), 0.939 [0.921–0.955] (240 s) and 0.954 [0.939–0.966] (300 s) for ∆MCAv_T_/∆MAP_T_^DEC^. Still at 0.10 Hz, within‐duration ICCs and their 95% CIs were 0.804 [0.738–0.859] (60 s), 0.885 [0.848–0.915] (120 s), 0.923 [0.899–0.943] (180 s), 0.939 [0.920–0.955] (240 s) and 0.953 [0.939–0.966] (300 s) for %MCAv_T_/%MAP_T_^INC^, and 0.814 [0.751–0.865] (60 s), 0.901 [0.870–0.928] (120 s), 0.938 [0.918–0.958] (180 s), 0.953 [0.938–0.965] (240 s) and 0.962 [0.950–0.972] (300 s) for %MCAv_T_/%MAP_T_^DEC^.

Between‐duration ICCs are reported in Tables 4 and 5, denoting an increasing reliability with longer RSS durations. In fact, ICCs and their 95% CIs were all good to excellent (>0.75) from 120 s duration at 0.05 Hz for both ∆MCAv_T_/∆MAP_T_ and %MCAv_T_/%MAP_T_. At 0.10 Hz, all duration ICCs and their 95% CIs were good to excellent from 60 s at 0.10 Hz ∆MCAv_T_/∆MAP_T_ and %MCAv_T_/%MAP_T_.

## 4 DISCUSSION

The results of this secondary analysis, completed on the largest sample size of healthy able‐bodied participants to date, are consistent with our previous findings reporting a directional sensitivity at 0.10 Hz but not 0.05 Hz, using RSS (Abbariki et al., 2022; Labrecque et al., 2022; Labrecque, Smirl, et al., 2021; Roy et al., 2022) or oscillatory lower‐body negative pressure (Labrecque et al., 2024). The novel findings of this study are as follows: (1) ∆MCAv_T_/∆MAP_T_ and %MCAv_T_/%MAP_T_ are capable of identifying directional sensitivity with shorter RSS durations; and (2) the reliability of ∆MCAv_T_/∆MAP_T_ and %MCAv_T_/%MAP_T_ remains with shorter RSS durations. Although all efforts should be made to record the reference standard of 300 s, it is possible to obtain reliable data from recordings of ≥180 s in young and healthy participants.

### 4.1 Directional sensitivity in the cerebral pressure–flow relationship using shorter durations of RSS

As expected, our analysis revealed that there is a directional sensitivity within the cerebrovasculature at 0.10 Hz, but not at 0.05 Hz, and these findings remained when shortening the duration of the RSS manoeuvre. At 0.05 Hz, multiple comparisons indicated that a minimum recording duration of 120 s should be used to obtain similar results to the 300 s reference standard when calculating ∆MCAv_T_/∆MAP_T_ and %MCAv_T_/%MAP_T_. At 0.10 Hz, multiple comparisons indicated that a minimum recording duration of 120 s for ∆MCAv_T_/∆MAP_T_ and 180 s for %MCAv_T_/%MAP_T_ should be used to obtain similar results compared to the 300 s reference standard. Considering the collective and comprehensive nature of these results, we recommend that recordings of ≥180 s of RSS are necessary to perform directional sensitivity analyses with confidence using our ratio metric.

Interestingly, we noted a greater variability (Figures 2, 3, 4, 5, 6, 7) of ∆MCAv_T_/∆MAP_T_ and %MCAv_T_/%MAP_T_ during INC. Concordantly, multiple comparisons revealed differences between shorter durations and the 300 s reference standard during INC, but not DEC. This indicates that the relationship between the rate of change in MCAv and MAP is more variable when MAP increases acutely than when MAP decreases acutely. A potential explanation for this discrepant finding could be that there are different mechanisms that regulate CBv in response to elevations and reductions in MAP. For instance, different activation of cerebral sympathetic nervous activity in response to acute increases versus decreases in MAP has been demonstrated in anaesthetized lambs (Cassaglia et al., 2008). Different myogenic control (Hamner & Tan, 2014; Tan et al., 2013) and venous outflow mechanisms (Donnelly et al., 2016; Wilson, 2016) could also potentially explain these different responses, although it remains to be determined clearly. Collectively, these mechanisms would be capable of providing additional buffering capacity against potential surges and increases in cerebral blood flow and would limit augmentations in intracranial pressure according to the Monro–Kellie doctrine (Brassard et al., 2021, 2023).

It was previously hypothesized that too small a sample size could explain why we do not observe a directional sensitivity at 0.05 Hz using our analytical approach (Panerai, 2023). However, the present study, including 99 participants, clearly demonstrates that this is not the case. We believe that differences in methodological and analytical techniques would explain why we do not observe a directional sensitivity at 0.05 Hz, whereas others do (Barnes et al., 2018; Panerai et al., 2018). For instance, the RSS protocol used by Panerai's group differs from ours, in that they asked their participants to adjust the depth of their squats. In addition, the autoregulation index analysis they used requires consideration of baseline resting values, which do not reflect the MAP and MCAv changes between minimums and maximums (Labrecque, Smirl, et al., 2021).

### 4.2 Reliability of ∆MCAv_T_/∆MAP_T_ and %MCAv_T_/%MAP_T_ using shorter durations of RSS

Whether looking at Bland–Altman plots, CoVs or ICCs, the reliability of ∆MCAv_T_/∆MAP_T_ and %MCAv_T_/%MAP_T_ improved with increasing RSS duration at both RSS frequencies. More specifically, from 180 s of recording onwards, Bland–Altman plots were considerably narrowed (Figures 4 and 5), all averaged CoVs were <5%, and all ICCs and their 95% CIs were >0.90. Therefore, to be confident in obtaining reliable averaged values of ∆MCAv_T_/∆MAP_T_ and %MCAv_T_/%MAP_T_ either during INC or DEC in MAP at both 0.05 and 0.10 Hz, we recommend that ≥180 s of continuous RSS recordings should be used with our ratio metric. Two other dCA studies have examined the possibility of shortening the duration of RSS using TFA metrics (Barnes et al., 2018; Burma, Miutz et al., 2021). Barnes et al. (2018) established that three RSS manoeuvres (60 s duration) were sufficient to obtain a coherence of >0.90 and therefore produce valid TFA estimates. However, their figure 5b shows that coherence becomes less variable between participants with a higher number of RSS (i.e., from 9 to 15 RSS). For their study specifically, it is important to mention that participants were asked to bend their knees as low as they were able for 300 s. This is a major difference from the 90° knee angle used by Burma, Miutz, et al. (2021) and in the present analysis. Allowing the participants to choose their squat depth has the potential to increase the variability of the haemodynamic response and is effectively apparent when we compare the CoVs calculated by Barnes et al. (2018) for TFA gain (∼20%) with those from Burma, Miutz, et al. (2021) (all <10%). Importantly, the standardization of RSS among participants should be ensured in reliability studies, which would enhance the possibility of comparing studies. Burma, Miutz, et al. (2021) went slightly further in their analyses by controlling for covariates ($P_{\mathrm{ET},CO_{2}}$, MAP, respiratory rate, etc.). These investigators concluded that RSS duration could be reduced to 240 s, and even 180 s, if covariates were controlled for, to produce reliable TFA estimates. These results support our recommendation of completing ≥180 s of RSS, because even if we did not correct for covariates, our reliability measures were high when comparing 180 and 300 s RSS durations. Nonetheless, we should be cautious when comparing these two studies (Barnes et al., 2018; Burma, Miutz, et al., 2021) with ours because TFA does not take the direction of MAP changes into consideration.

Interestingly, we denoted a greater variability and slightly lower reliability (Tables 4 and 5) for ∆MCAv_T_/∆MAP_T_ and %MCAv_T_/%MAP_T_ at 0.05 versus 0.10 Hz. Yet, Burma, Miutz, et al. (2021) identified fewer sources of confounding variables at 0.05 than at 0.10 Hz. This greater variability observed in the present analysis could be explained by the notion that the 0.10 Hz frequency includes more oscillations for the same RSS duration. Therefore, the impact of extreme values is diluted.

Of note, some of our reliability estimations were elevated even for the 60 s duration, particularly at 0.10 Hz. This could be explained by the notion that after visual inspection, we removed the first(s) oscillation(s), which were unstable, at the beginning of each recording. Concordantly, Panerai et al. (2018) also demonstrated that the first RSS manoeuvre was different from the subsequent ones. The removal of unstable oscillations should be considered when planning reliability studies using RSS by ensuring a longer recording duration than needed.

### 4.3 Methodological considerations

There are some limitations to this study that should be discussed further. Only healthy young males and females were included in the present analysis. Accordingly, results cannot be extended to older or clinical populations. Give that the ratio metric has been measured only in the MCA, these results cannot apply to other cerebral vessels. Moreover, MCAv was estimated with transcranial Doppler ultrasound, which is considered representative of blood flow if the diameter of the artery remains stable (Ainslie & Hoiland, 2014). Also, it is important to note that the within‐ and between‐session reproducibility of the metric applied on shorter durations has not been demonstrated. Caution should therefore be exercised if such comparisons were to be completed.

Variations in $P_{\mathrm{ET},CO_{2}}$ levels have been associated with changes in the diameter or cross‐sectional area of the MCA (Al‐Khazraji et al., 2019; Verbree et al., 2014). In the present study, $P_{\mathrm{ET},CO_{2}}$ during RSS at 0.10 Hz was different from baseline, and the averaged $P_{\mathrm{ET},CO_{2}}$ during RSS was different between frequencies. However, the aim of the present study was not to compare frequencies. Also, $P_{\mathrm{ET},CO_{2}}$ was lower for 60, 120 and 180 s durations when compared with the reference 300 s duration at both frequencies. However, we believe these differences had a minimal impact on our results because absolute differences between $P_{\mathrm{ET},CO_{2}}$ of shorter durations and the 300 s reference were all <1 Torr (Table 3). Interestingly, it was previously observed that there was no change in $P_{\mathrm{ET},CO_{2}}$ between the squatting and standing phases (Panerai et al., 2018). This is of major importance considering that our directional sensitivity analysis compares INC and DEC in MAP during RSS. Unfortunately, using our methodological approach, it is impossible for us to provide exact and synchronized measures of $P_{\mathrm{ET},CO_{2}}$ during INC and DEC in MAP independently. Of note, although at rest and using a different analytical method, it was previously demonstrated that hypercapnia does not influence the strength of directional sensitivity (Panerai et al., 2023). Further research will be necessary, in which $P_{\mathrm{ET},CO_{2}}$ is clamped at baseline levels (or during hypocapnia and hypercapnia), to examine the impact of CO_2_ on the directional sensitivity of the cerebral pressure–flow relationship.

**TABLE 3:** Averaged absolute changes, time intervals, and rate of change in mean arterial pressure and middle cerebral artery mean blood velocity for all repeated squat–stand durations at 0.05 and 0.10 Hz.

Parameter		60 s	120 s	180 s	240 s	300 s	Duration	Direction	Interaction
**0.05 Hz**									
∆MAP 0.05 Hz, mmHg	INC	40 ± 13^a^	41 ± 14^a^	41 ± 14^a^	42 ± 14^a^	42 ± 14	<0.0001	0.021	0.017
	DEC	40 ± 14^a^	41 ± 14^a^	41 ± 14^a^	42 ± 14^a^	42 ± 14			
∆MCAv 0.05 Hz, cm/s	INC	33 ± 13^a^	34 ± 13^a^	35 ± 13^a^	35 ± 13^a^	35 ± 14	<0.0001	<0.001	<0.0001
	DEC	33 ± 12^a^	34 ± 13^a^	34 ± 13^a^	35 ± 13^a^	35 ± 13			
∆Time for MAP change 0.05 Hz, s	INC	9.0 ± 1.6	9.2 ± 1.3	9.2 ± 1.2	9.2 ± 1.1	9.2 ± 1.0	0.744	<0.0001	0.332
	DEC	10.9 ± 1.4	10.8 ± 1.2	10.8 ± 1.2	10.8 ± 1.1	10.8 ± 1.0			
∆Time for MCAv change 0.05 Hz, s	INC	9.5 ± 1.7^a^	9.4 ± 1.5	9.3 ± 1.2	9.3 ± 1.2	9.2 ± 1.2	0.982	<0.0001	0.006
	DEC	10.5 ± 1.4	10.6 ± 1.2	10.7 ± 1.1	10.7 ± 1.1	10.7 ± 1.1			
Rate of change in MAP 0.05 Hz, mmHg/s	INC	4.7 ± 1.9	4.8 ± 1.9	4.8 ± 1.9	5.1 ± 2.5	5.1 ± 2.3	<0.001	<0.0001	0.415
	DEC	3.8 ± 1.2	3.9 ± 1.3	4.0 ± 1.3	4.0 ± 1.3	4.0 ± 1.3			
Rate of change in MCAv 0.05 Hz, cm/s/s	INC	3.6 ± 1.3	3.7 ± 1.2	3.8 ± 1.2	3.9 ± 1.3	4.0 ± 1.3	<0.0001	<0.0001	0.056
	DEC	3.3 ± 1.3	3.3 ± 1.4	3.4 ± 1.4	3.4 ± 1.4	3.5 ± 1.4			
**0.10 Hz**									
∆MAP 0.10 Hz, mmHg	INC	34 ± 10^a^	36 ± 10^a^	37 ± 11^a^	37 ± 11^a^	38 ± 11	<0.0001	0.181	<0.0001
	DEC	34 ± 10^a^	36 ± 10^a^	37 ± 11^a^	37 ± 11^a^	38 ± 11			
∆MCAv 0.10 Hz, cm/s	INC	30 ± 10	32 ± 11	33 ± 11	33 ± 11	34 ± 11	<0.0001	0.011	0.162
	DEC	30 ± 10	32 ± 11	33 ± 11	33 ± 11	34 ± 11			
∆Time for MAP change 0.10 Hz, s	INC	4.7 ± 0.4^a^	4.8 ± 0.4	4.8 ± 0.4	4.8 ± 0.4	4.8 ± 0.4	0.561	<0.0001	<0.001
	DEC	5.3 ± 0.4^a^	5.2 ± 0.4	5.2 ± 0.4	5.2 ± 0.4	5.2 ± 0.4			
∆Time for MCAv change 0.10 Hz, s	INC	5.1 ± 0.6	5.1 ± 0.6	5.1 ± 0.5	5.1 ± 0.5	5.1 ± 0.5	<0.001	0.181	0.957
	DEC	5.0 ± 0.7	4.9 ± 0.6	4.9 ± 0.5	4.9 ± 0.5	4.9 ± 0.5			
Rate of change in MAP 0.10 Hz, mmHg/s	INC	7.6 ± 3.2	7.9 ± 2.8	8.0 ± 2.7	8.1 ± 2.9	8.2 ± 2.8	<0.0001	<0.0001	0.0874
	DEC	6.6 ± 1.9	7.0 ± 1.9	7.2 ± 2.0	7.3 ± 2.0	7.4 ± 2.0			
Rate of change in MCAv 0.10 Hz, cm/s/s	INC	6.2 ± 2.4	6.6 ± 2.5	6.8 ± 2.6	6.9 ± 2.6	7.0 ± 2.7	<0.0001	0.793	0.976
	DEC	6.2 ± 2.0	6.7 ± 2.1	6.9 ± 2.2	7.0 ± 2.2	7.1 ± 2.3			

*Note*: Values are the mean ± SD. *n* = 99. Abbreviations: DEC, decrease; INC, increase; MAP, mean arterial pressure; MCAv, middle cerebral artery mean blood velocity.

^a^ Value different from 300 s.

**TABLE 4:** Coefficient of variation and intraclass correlation coefficient of ∆MCAv_T_/∆MAP_T_ comparing each RSS duration to the 300 s reference standard at 0.05 and 0.10 Hz.

	RSS duration	CoV (median)	ICC
0.05 Hz INC	60 vs. 300 s	11.5 ± 9.1 (9.1)	0.736 (0.631–0.815)
	120 vs. 300 s	6.7 ± 5.9 (4.5)	0.924 (0.889–0.948)
	180 vs. 300 s	3.9 ± 3.5 (2.8)	0.972 (0.958–0.981)
	240 vs. 300 s	2.3 ± 2.9 (1.7)	0.988 (0.982–0.992)
0.05 Hz DEC	60 vs. 300 s	9.9 ± 8.6 (7.6)	0.775 (0.682–0.843)
	120 vs. 300 s	6.1 ± 5.7 (4.3)	0.900 (0.855–0.932)
	180 vs. 300 s	3.3 ± 2.6 (3.0)	0.969 (0.955–0.979)
	240 vs. 300 s	1.7 ± 1.6 (1.3)	0.991 (0.986–0.994)
0.10 Hz INC	60 vs. 300 s	7.0 ± 5.6 (5.7)	0.886 (0.835–0.922)
	120 vs. 300 s	4.5 ± 4.0 (3.9)	0.947 (0.923–0.964)
	180 vs. 300 s	2.8 ± 2.3 (2.4)	0.979 (0.968–0.986)
	240 vs. 300 s	1.3 ± 1.2 (1.0)	0.995 (0.992–0.996)
0.10 Hz DEC	60 vs. 300 s	6.7 ± 5.1 (5.7)	0.886 (0.835–0.922)
	120 vs. 300 s	4.7 ± 3.5 (4.2)	0.947 (0.922–0.964)
	180 vs. 300 s	3.0 ± 2.3 (2.3)	0.977 (0.967–0.985)
	240 vs. 300 s	1.5 ± 1.2 (1.2)	0.994 (0.991–0.996)

*Note*: Values for CoV are mean ± SD, ICC (95% limits of agreement). *n* = 99.

Abbreviations: CoV, coefficient of variation; DEC, acute decreases in mean arterial pressure; ICC, intraclass correlation coefficient; INC, acute increases in mean arterial pressure; RSS, repeated squat–stand; ΔMCAv_T_/ΔMAP_T_, time‐adjusted ratio between middle cerebral artery mean blood velocity and mean arterial pressure.

**TABLE 5:** Coefficient of variation and intraclass correlation coefficient of %MCAv_T_/%MAP_T_ comparing each repeated squat–stand duration with the 300 s reference standard at 0.05 and 0.10 Hz.

		CoV (median)	ICC
0.05 Hz INC	60 vs. 300 s	11.9 ± 9.2 (10.3)	0.778 (0.687–0.846)
	120 vs. 300 s	7.2 ± 5.8 (5.7)	0.926 (0.891–0.949)
	180 vs. 300 s	4.0 ± 3.6 (3.2)	0.973 (0.960–0.982)
	240 vs. 300 s	2.2 ± 1.8 (1.8)	0.991 (0.986–0.994)
0.05 Hz DEC	60 vs. 300 s	10.7 ± 9.4 (7.2)	0.801 (0.717–0.862)
	120 vs. 300 s	6.3 ± 5.7 (4.5)	0.925 (0.891–0.949)
	180 vs. 300 s	3.6 ± 2.7 (3.2)	0.979 (0.968–0.986)
	240 vs. 300 s	1.9 ± 1.8 (1.4)	0.992 (0.989–0.995)
0.10 Hz INC	60 vs. 300 s	7.7 ± 6.5 (5.9)	0.872 (0.815–0.912)
	120 vs. 300 s	4.8 ± 4.5 (4.2)	0.944 (0.918–0.962)
	180 vs. 300 s	2.8 ± 2.6 (1.9)	0.977 (0.966–0.984)
	240 vs. 300 s	1.5 ± 1.4 (1.1)	0.994 (0.991–0.996)
0.10 Hz DEC	60 vs. 300 s	6.8 ± 5.6 (5.3)	0.907 (0.865–0.937)
	120 vs. 300 s	5.2 ± 4.1 (4.4)	0.953 (0.931–0.968)
	180 vs. 300 s	3.4 ± 2.7 (2.7)	0.980 (0.970–0.986)
	240 vs. 300 s	1.6 ± 1.3 (1.3)	0.995 (0.993–0.997)

*Note*: Values for CoV are the mean ± SD, ICC (95% limits of agreement). *n* = 99.

Abbreviations: CoV, coefficient of variation; DEC, acute decreases in mean arterial pressure; INC, acute increases in mean arterial pressure; ICC, intraclass correlation coefficient; RSS, repeated squat–stand; %MCAv_T_/%MAP_T_, time‐adjusted ratio between middle cerebral artery mean blood velocity and mean arterial pressure in relative term.

In the present investigation, females were prescribed oral contraceptives (*n* = 2) or were wearing an intrauterine device (*n* = 1) or were tested in days 1–10 of their menstrual cycle. Given that this study was a within‐participant comparison of various RSS durations and that no biological sex comparison was completed, we consider that it had a minor impact on our results.

Another potential issue that could affect our directional sensitivity findings is that we did not consider the duration of each postural change (i.e., from standing to squatting and vice versa). Although the RSS transitions were tightly set to last either 5 or 10 s, we acknowledge that the time taken to complete the movement might have varied between each squat–stand transition and between participants. However, we consider that the adjustment for time intervals corrects for potential differences in the timing of postural change.

In addition, our results demonstrate that the rate of change in MAP is different between INC and DEC, making the MAP stimulus for MCAv changes different between the directions. It was suggested that the rate of change in MAP could be the dominant factor explaining the presence of an asymmetry in the cerebral pressure–flow relationship (Panerai et al., 2018). However, it is difficult to establish whether this is inherent to cardiovascular control of blood pressure or whether we could control for it externally by adapting the speed of RSS transition, for instance.

Another issue, which could potentially contribute to the differences reported at 0.05 and 0.10 Hz, is the different number of transitions included in the comparison between each RSS duration. Although slightly lower at 0.05 Hz, reliability measures are comparable between frequencies. Nevertheless, having double the number of repetitions for 0.10 Hz RSS could explain why reliability remains better at shorter durations compared with 0.05 Hz RSS. To evaluate the potential impact of this issue, future studies should record the same number of repetitions at each RSS frequency, meaning that longer RSS durations would have to be completed at 0.05 Hz. Therefore, both the same RSS duration and number could be compared for each RSS frequency.

Also, our analyses could be influenced by the lack of independence between durations, where shorter RSS durations are included in longer RSS durations. Future studies should compare separate recordings of each RSS duration. Likewise, it would also be of great interest to compare the first 60 and 120 s with the last 60–120 s.

It is also important to note that the ICC has limits, such as being influenced by a heterogeneous sample (Hartmann et al., 2023). However, in addition to having a relatively homogeneous sample of participants, we are confident our ICC values provide a good estimate of reliability, because Bland–Altman plots and CoV also indicate a very good level of reliability.

Finally, the proposed directional sensitivity metric has not yet been validated because there is no gold standard with which to compare it. Moreover, the metric has not been compared between healthy participants and a diseased population known to have impaired dCA.

### 4.4 Perspectives

These findings are of great importance in the broader field of cerebral autoregulation and, more specifically, for investigations into the hysteresis nature of the cerebral pressure–flow relationship. The present findings will help future studies to be designed better and potentially allow clinical populations to be studied when directional sensitivity assessments are performed. Of course, a similar assessment should be completed in clinical populations. Importantly, it will also allow researchers to use shortened data collection when it is not possible to collect an entire 300 s duration continuous recording. Although the present analysis establishes the basis for using shorter recordings when assessing directional sensitivity with RSS, many questions remain to be answered within this field. For example, will similar results be observed for investigations completed with passively induced blood pressure oscillations using oscillatory lower‐body negative pressure or within clinical populations? Methodological aspects such as controlling for covariates and comparing independent sections of different recording lengths are also worth investigating. Also, future studies aiming at evaluating the reliability of directional sensitivity metrics should be planned thoroughly in order that various recording durations would be completed, with a concern for having the same number of repetitions for both RSS frequencies. For instance, to compare 30 squat–stand repetitions between both RSS frequencies, 0.05 and 0.10 Hz RSS would need to have durations of 10 and 5 min, respectively. The between‐session  reproducibility should also be evaluated in future studies.

## 5 CONCLUSION

This analysis aimed to evaluate the ability of a ratio metric to identify directional sensitivity for shorter RSS durations and to establish its reliability over shorter RSS durations compared with the 300 s standard. Our results show that shorter recording durations of RSS can be used to quantify directional sensitivity in the cerebral pressure–flow relationship, although recordings <180 s should not be used. Specifically, our findings suggest that 240 and 180 s recording lengths of forced MAP oscillations can be used to quantify cerebral pressure–flow relationship directional sensitivity in both INC and DEC, which could be beneficial when examining clinical populations or individuals with low cardiorespiratory fitness who might be unable to sustain the full 5 min of RSS owing to high physical demand. Nonetheless, it is still recommended that researchers aim to make all efforts to record ≥5 min of high‐quality RSS‐induced oscillations.

## AUTHOR CONTRIBUTIONS

Conception or design of the work: Lawrence Labrecque and Patrice Brassard. Acquisition or analysis or interpretation of data for the work: Lawrence Labrecque, Marc‐Antoine Roy, Shahrzad Soleimani Dehnavi, Mahmoudreza Taghizadeh, Joel S. Burma, Jonathan D. Smirl and Patrice Brassard. Drafting the work or revising it critically for important intellectual content: Lawrence Labrecque, Marc‐Antoine Roy, Shahrzad Soleimani Dehnavi, Mahmoudreza Taghizadeh, Joel S. Burma, Jonathan D. Smirl and Patrice Brassard. Final approval of the version to be published: Lawrence Labrecque, Marc‐Antoine Roy, Shahrzad Soleimani Dehnavi, Mahmoudreza Taghizadeh, Joel S. Burma, Jonathan D. Smirl and Patrice Brassard. Agreement to be accountable for all aspects of the work in ensuring that questions related to the accuracy or integrity of any part of the work are appropriately investigated and resolved: Lawrence Labrecque, Marc‐Antoine Roy, Shahrzad Soleimani Dehnavi, Mahmoudreza Taghizadeh, Joel S. Burma, Jonathan D. Smirl and Patrice Brassard. All persons designated as authors qualify for authorship, and all those who qualify for authorship are listed.

## CONFLICT OF INTEREST

None declared.

## Data Availability

The data supporting the findings from this manuscript are available upon reasonable request to the corresponding author.
